# Predicting tree pollen season start dates using thermal conditions

**DOI:** 10.1007/s10453-014-9329-3

**Published:** 2014-02-20

**Authors:** Dorota Myszkowska

**Affiliations:** Department of Clinical and Environmental Allergology, Jagiellonian University Medical College, Śniadeckich 10, 31-531 Kraków, Poland

**Keywords:** *Alnus*, *Corylus*, *Betula* pollen seasons, Mean and maximum temperature fluctuations, Logistic regression, Predictive models

## Abstract

Thermal conditions at the beginning of the year determine the timing of pollen seasons of early flowering trees. The aims of this study were to quantify the relationship between the tree pollen season start dates and the thermal conditions just before the beginning of the season and to construct models predicting the start of the pollen season in a given year. The study was performed in Krakow (Southern Poland); the pollen data of *Alnus*, *Corylus* and *Betula* were obtained in 1991–2012 using a volumetric method. The relationship between the tree pollen season start, calculated by the cumulated pollen grain sum method, and a 5-day running means of maximum (for *Alnus* and *Corylus*) and mean (for *Betula*) daily temperature was found and used in the logistic regression models. The estimation of model parameters indicated their statistically significance for all studied taxa; the odds ratio was higher in models for *Betula*, comparing to *Alnus* and *Corylus*. The proposed model makes the accuracy of prediction in 83.58 % of cases for *Alnus*, in 84.29 % of cases for *Corylus* and in 90.41 % of cases for *Betula*. In years of model verification (2011 and 2012), the season start of *Alnus* and *Corylus* was predicted more precisely in 2011, while in case of *Betula,* the model predictions achieved 100 % of accuracy in both years. The correctness of prediction indicated that the data used for the model arrangement fitted the models well and stressed the high efficacy of model prediction estimated using the pollen data in 1991–2010.

## Introduction

Temperature is one of the major environmental factors that determines the readiness of plants to flower. In temperate climate, many species need a period of exposure to low, but not freezing temperature to acquire reproductive competence and an ability to flower (Dahl et al. 2013). For trees, especially those pollinating at the beginning of the year, thermal conditions and critical photoperiod following the time of the chilling control the start of flowering and leaf bud burst (Sofiev et al. 2009).

The different phenomena connected with the relationship between meteorological elements and plant behaviour, including the pollen occurrence in the atmosphere, are used to construct the mathematical models predicting the pollen season start. The *observation*-*based models* refer to the relationship between the pollen season start day and many meteorological elements, such as air temperature, rainfall, sunshine and snow cover occurring both before the pollen season and in the preceding season (Emberlin et al. 1993; Norris-Hill 1998; Corden et al. 2000; Adams-Groom et al. 2002; Rodriguez-Rajo et al. 2004). These models are constructed without knowledge of the sources, emission or calculations of diffusion (Norris-Hill 1995). In contrast, *process*-*based* models refer to a given relationship between analysed variables. In this group of models, thermal-time models are most often used in phenological and aerobiological studies. These models work on the assumption that the speed of plant development depends on air temperature, which influences physiological processes like flower bud opening. Some papers on the thermal conditions required for the pollen season start dates of *Corylus* (Frenguelli et al. 1991; Frenguelli and Bricchi 1998), *Alnus* (Jato et al. 2000; Clot 2001; Rodriguez-Rajo et al. 2006; Emberlin et al. 2007) and *Betula* (Linkosalo et al. 2010) were published.

A review of threshold temperatures required by early-pollinating trees was presented by Dahl et al. (2013). Some controversies are related to the introduction of the time of absolute dormancy into the most sophisticated models. In temperate climates, the dormancy period of trees ends at the end of December or in January. Temperature, relative humidity and the number of days with sub-zero temperatures were reported as the main factors determining the start of *Corylus* pollen seasons in Sosnowiec (Silesia region, southern Poland) (Dąbrowska-Zapart 2008), while Piotrowska and Kaszewski (2009) pointed out the influence of accumulated 5-day running mean temperatures before the beginning of the *Corylus* pollen season in Lublin (eastern Poland). Phenological and aerobiological observations performed by Puc and Kasprzyk (2013) in Szczecin (Northwestern Poland) and Rzeszów (Southeastern Poland) indicated that hazel and alder trees flowered earlier in stands located in places exposed to sunlight and sheltered from the wind.

The previous analyses performed in Krakow showed that temperature during the last 10 days of February and relative humidity at the beginning of April were the best at explaining variations in *Betula* pollen season start dates and were included into multiple regression models as independent variables (Myszkowska 2013). In *Alnus* and *Corylus* pollen seasons, a strong negative correlation between the season start and mean temperature in January and mean temperatures during the 10 days just before the onset of the season was found by Myszkowska et al. (2006), while the paper by Piotrowicz and Myszkowska (2006) showed a statistically significant correlation between start dates of *Corylus* pollen seasons and the beginning of thermal early spring (temperature >0 °C).

Many aerobiological studies are focused on predicting the season start for medical purposes (Burr 1999; Chappard et al. 2004; Chuine and Belmonte 2004). In Poland, the early-pollinating trees, including *Alnus*, *Corylus* and (a bit later) *Betula,* are of great importance from a medical point of view. The analyses made by Burbach et al. (2009) showed that the clinically relevant sensitisation rate of *Alnus*, *Corylus* and *Betula* in Poland ranged from 59.6 % (both for *Alnus* and *Corylus*) to 70.8 % for *Betula*. The study conducted by Samoliński et al. (2009) in a group of 20,454 subjects in Poland indicated that the allergens of these tree taxa are responsible for allergic rhinitis symptoms generated in sensitive patients in spring in Poland. The construction of simple models based on thermal conditions has a practical application in allergology, e.g. to estimate the beginning of the allergen immunotherapy in patients sensitive to tree pollen allergens, especially in case of a preseasonal treatment.

The aims of this study were to examine the relationship between start dates of tree pollen seasons and thermal conditions just before the season and to construct models predicting the start of the pollen season in a given year.

## Materials and methods

### Study site and climate

The study was performed in Krakow (50°3′41″N, 19°56′18″E) (Southern Poland), one of the largest cities in Poland (Report on city conditions 2011) (http://www.bip.krakow.pl). To the north of Krakow, farmland with small forest communities occur. To the south and east, there are roughly equal areas of farmland and forests. To the west of the city, forest communities prevail. In Krakow, forests cover 4.38 % of the total city area (1,431 ha). Krakow (and Poland in general) is influenced by air masses of polar–maritime origin coming from over the Northern Atlantic, which bring increased cloudiness, precipitation, warming and thaw in winter and increased cloudiness, rainfall and chilling in summer. The mean air temperature in Krakow in the twentieth century was 8.7 °C; 2000 was the hottest year (11 °C) (Piotrowicz 2007). The coldest month is January and the hottest July (with monthly temperature of −2.1 and 18.9 °C, respectively). Sunshine duration per day is 3.9 h although from April to September it is 5.7 h (Woś 1999). Annual precipitation is approximately 700 mm. The highest rainfall level is recorded in summer (June, July and August). Annual humidity is 79 %, and winds from a westerly direction prevail. In Krakow, a heat island occurs with intensity in the city centre reaching 1.2 °C on average. The heat island is responsible for a change in the thermal season duration in the year. Summer in the city centre is longer by 25 days, and winter is shorter by 23 days than in suburban areas. Higher temperature in the city centre causes a longer vegetation season.

### Studied taxa

All of the described taxa belong to the Betulaceae family (APGII 2003; APGIII 2009). *Alnus glutinosa* (L.) Gaertn., *A. incana* (L.) Moench and a shrub *Alnus viridis* (Chaix) DC. in Lam. & DC. belonging to genus *Alnus* Miller occur in Poland. *A. glutinosa* is commonly found throughout the country, in wet forests, on the banks of streams, in river valleys and on lake shores, while *A. incana* is rather common in the southern part of Poland. *A. viridis* is found in the western part of the Bieszczady Mountains, from an altitude of 600 m a.s.l. up to summits (Seneta and Dolatowski 2007). In Poland, *A. glutinosa* usually starts flowering in the third decade of March or at the beginning of April, while the beginning of *A. incana* flowering is noted several days to three weeks earlier than *A. glutinosa* flowering. *A. viridis* starts flowering even at the end of May or at the beginning of June.

In Krakow, *A. glutinosa* is the most common *Alnus* species, especially in the southern part of the city and close to the city border in the north. Many *A. glutinosa* sites are observed in the vicinity of Krakow, particularly in the northwest part of the city and more commonly to the south of Krakow (Zając and Zając 2006).

The genus *Corylus* L. is commonly found throughout the whole of Poland. *C. avellana* L. occurs as a high shrub (4 m) or less often as a low tree. There are fewer *Corylus* sites within Krakow compared to outside of the city, the exception being the northeastern part of the Krakow (Zając and Zając 2006). *Corylus* is planted as an ornamental tree in gardens.

Generally speaking, seven species of *Betula* occur in Poland. The most frequent are *B. pendula* Roth (*B. verrucosa* Ehrh.) and *B. pubescens* Ehrh. The most common species is *B. pendula*, which occurs throughout the country. In Krakow and its close surroundings, *B. pendula* dominates, while *B. pubescens* occasionally occurs in the Niepołomice Forest and towards the northwestern part of Krakow (Zając and Zając 2006). *B. pendula* starts flowering 10–12 days earlier than *B. pubescens* (Suszka 1979).

### Pollen data collection

The studied taxa were selected on the basis of high allergenicity and the common occurrence in the city and in its close vicinity. Pollen data were collected using the volumetric method in 1991–2012. Two spore traps of the Hirst design (Hirst 1952) were used (Seven Day Recording Volumetric Spore Trap, Burkard Company in 1991–2003 and VPPS 2000, Lanzoni Ltd. in 2004–2010). The samplers were located on the roof of the Collegium Sniadeckiego building (Institute of Botany, Jagiellonian University) in the city centre 20 m above ground level. Pollen grains were sucked onto a rotating drum covered by transparent (Melinex) tape.

The arrangement of microscopic slides was made according to Jäger et al. (1995). The daily samples were examined using a light microscope at 400 × magnification. Pollen grains were counted along 4 longitudinal transects in 2000–2012, and earlier in 1991–1999, the 12 traverse transects method was employed. Both methods are recommended by the European Aerobiology Society rules of quality control (www.ean.polleninfo.org). The use of both longitudinal and latitudinal (transverse) traverses has been shown to produce comparable results when similar percentages of the slide are examined (Comtois et al. 1999; Carinanos et al. 2000). However, Kapyla and Penttinen (1981) stated that traverses along the length of the slide may give unreliable estimates because of the irregular transverse variation in the deposition of particles on the tape. The authors recommended that whole width of the tape should be studied because considerable amounts of particles were also found outside the 14-mm-wide “effectively collecting area” below the orifice (Kapyla and Penttinen 1981). Cotos-Yáñez et al. (2013) also reported that the distribution of the variable number of pollen grains over longitudinal and transversal traverses is not uniform.

Pollen season start was calculated using the pollen grain sum method of 5 grains cumulated since January 1 for *Alnus* and *Corylus*, while the pollen grain sum method of 15 grains cumulated since March 1 in case of *Betula* (about 40 days before the mean season start). The sum cumulative method was recommended by Adams-Groom et al. (2002) and Rodriguez-Rajo et al. (2006).

The seasons were divided into three groups according to the season start day calculated since January 1 and described as follows: for *Alnus*—early season (E) 12–36th day, moderate season (M) 37–60th day, late season (L) 61st–84th day; for *Corylus*—early season (E) 11–36th day, moderate season (M) 37th–61st day, late season (L) 62nd–85th day; and for *Betula*—early season (E) 87–95th day, moderate season (M) 96–104th day, late season (L) 105–113th day. Time range from the latest start of the pollen season to the earliest one was divided into three periods, where median of each period was 24.

### Meteorological data and study idea

The meteorological data were provided by the Research Station of the Dept. of Climatology, Institute of Geography and Spatial Management, Jagiellonian University (50°04′N, 19°58′E, h 206°m a.s.l.), located in the immediate vicinity of the monitoring site. Minimum, maximum and mean daily temperature as initial data were taken. Mean daily temperature was calculated using the formula: (*t*_max_ + *t*_min_ + *t*_6_ + *t*_18_)/4 (*t*_6_—temperature at 6 a.m. UTC, *t*_18_—temperature at 6 p.m. UTC).

Five-day running means of different temperature parameters were calculated because it was found that these variables were the best at describing the relationship between start dates of each studied taxon and thermal conditions. These running means were counted since January 1 to the day calculated as the season start for *Alnus* and *Corylus* and since March 5 in case of *Betula*. Running means including 5 consecutive values of daily mean temperature were named as a 5-day temperature mean. A 5-day mean temperature on January 1 was counted from December 28 to January 1 inclusive, and a 5-day mean temperature on March 5 was counted from March 1 to March 5 inclusive.

**Fig. 1:**
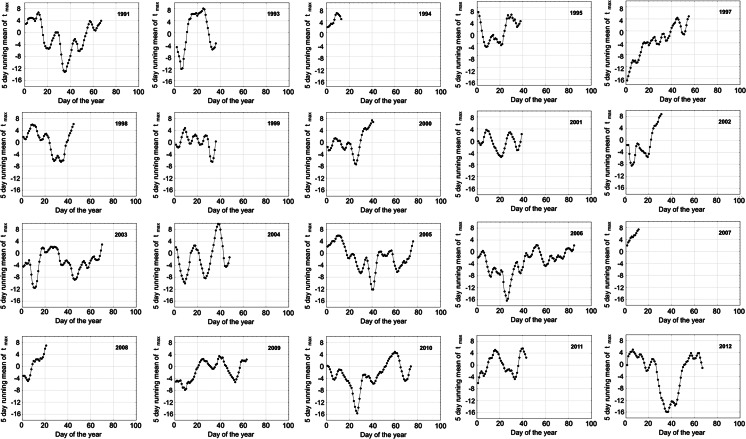
The scatter plots of seasonal fluctuations of 5-day running mean of maximum temperature (as described in Sect. 2) before the *Corylus* pollen seasons in Krakow in 1991–2012. Years of 1992 and 1996 were excluded from the analyses. The cubic spline was used to smooth the curve

**Fig. 2:**
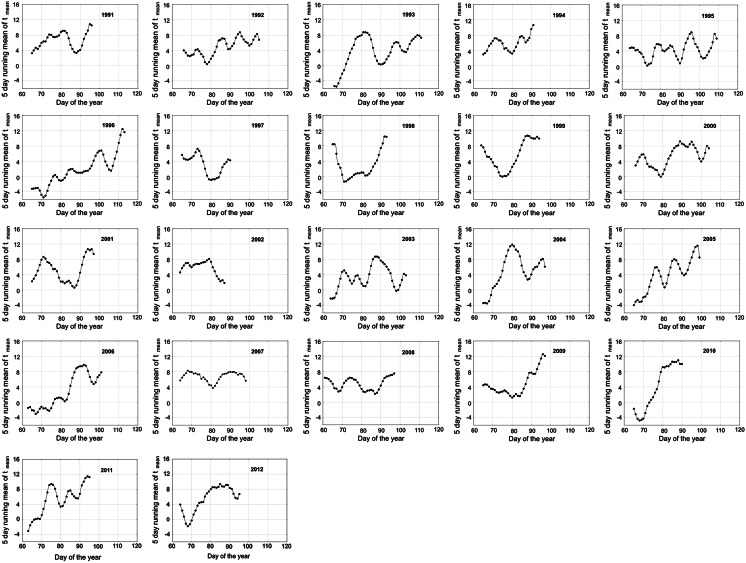
The scatter plots of seasonal fluctuations of 5-day running mean of maximum temperature (as described in Sect. 2) before the *Betula* pollen seasons in Krakow in 1991–2012. The cubic spline was used to smooth the curve

The aim of the statistical analysis was to find a model, which would be able to predict a day of the tree pollen season start with a high accuracy. The analysis was based on the changes in temperature before the pollen season. Within a 20-year aerobiological monitoring, the increase in temperature followed by the temperature decrease just before a season was noted in majority of the studied years (Figs. 1, 2). It was assumed that this relatively short time period of temperature decrease followed by an increase in temperature (described as the “local minimum” from now on) could be used to indicate an approaching pollen season. This “local minimum” will consequently be used analogically to the mathematical terminology (http://mathworld.wolfram.com).

The author’s intention was to find such a “local minimum”, which could be an optimal indicator. Looking at the Figs. 1 and 2 with 5-day temperature fluctuations, we can see the periods of temperature increase and decrease depending on a given day. The proposed methodology, based on temperature fluctuations just before a season, was named in a working version “hills and dales theory”. A day described as the “local minimum” was accepted as a day preceded by at least two days, when the decrease in a 5-day running mean temperature was noted and then followed by at least two days, when the increase in a 5-day running mean temperature was observed. Local minima that occurred less than three days before the season start for each taxon were not included in the analysis.

### Predictive models

The nonlinear logistic regression was used to find the probability of the pollen season start up to ten days from the “local minimum” calculated on the basis of 5-day running mean temperature. Whereby the pollen season start is a dichotomous variable that deals with only two possible values: “1” (YES), if the season starts within ten days from the “local minimum”, and “0” (NO), if the season does not start in the time mentioned above.

In the case of *Alnus* and *Corylus* pollen seasons, the following variables were included in the models:

- a day calculated as a “local minimum” (consecutive day of a year)
- 5-day running mean of daily maximum temperature on the day of “local minimum”

In case of *Betula* pollen seasons, the following variables were included into a model:

- a day calculated as a “local minimum” (consecutive day of a year)
- 5-day running mean of daily mean temperature on the day of “local minimum”

The parameters of the logistic regression models were estimated by regression coefficients and odds ratio calculated as the ratio of probability that a given case does happen to a probability that a given case does not happen. The value of *χ* ^2^ statistics was used to estimate the goodness of model fit as one of the parts of the statistical procedure in the logistic regression (*p* < 0.05).

Among the constructed models, a 10-day model was selected as the most effective. The models assume that some case will happen when its probability is higher than 0.5. Presented models were constructed on the basis of pollen and meteorological data obtained in 1991–2010. Then, the models were verified with the data obtained in 2011 and 2012. The values of probability of the *Alnus* and *Corylus* pollen season start occurring, calculated using model parameters for critical 5-day running means of maximum temperature ranging from −6 to +4 °C, were graphically presented. The probability of the *Alnus* and *Corylus* pollen season start occurring at a critical temperature of 4 and −4 °C was also calculated. In the case of *Betula*, the probability of pollen season start occurring was presented at critical temperature of 2.4, 4.4 and 10.5 °C. Temperature values were chosen on the basis of the precise analyses of the thermal situation in the study years. The threshold values were indicated as the lowest or the highest temperature obtained in the analysed “local minimum”; in case of the *Betula* pollen season, the mean temperature was tested. The aim of this analysis was to test how prediction of the model behaves for threshold values.

Analyses were carried out, using the Statistica program version 9.0.

## Results

### Tree pollen season start dates

The pollen seasons of *Alnus* and *Corylus* tended to occur at roughly the same time (Table 1). The season starts of both taxa varied strongly from year to year, which makes these characteristics difficult to predict. The seasons started, on average, in the middle of February (46–47th day of the year). In the studied period, no seasons described as early, moderate and late prevailed; the tendency towards earlier or later season start was not found.

**Table 1:** Descriptive statistics of tree pollen season starts in Krakow, in 1991–2010

Taxon	*Alnus*			*Corylus*			*Betula*		
Season	*Σ*5*	Date	Season type	*Σ*5*	Date	Season type	*Σ*15*	Date	Season type
1991	67	8/03	L	67	8/03	L	96	6/04	M
1992	–	–	–	–	–	–	105	14/04	L
1993	36	4/02	E	36	4/02	E	111	20/04	L
1994	12	12/01	E	13	13/01	E	91	1/04	E
1995	39	8/02	M	38	7/02	M	109	25/03	L
1996	–	–	–	–	–	–	113	23/04	L
1997	55	24/02	M	55	24/02	M	90	25/03	E
1998	43	12/02	M	45	16/02	M	93	3/04	E
1999	35	4/02	E	36	5/02	E	94	4/04	E
2000	40	9/02	M	41	10/02	M	104	13/04	M
2001	39	8/02	M	39	8/02	M	97	7/04	M
2002	25	25/01	E	31	31/01	E	87	28/03	E
2003	66	7/03	L	70	11/03	L	103	13/04	M
2004	51	20/02	M	48	17/02	M	97	6/04	M
2005	77	18/03	L	76	17/03	L	99	8/04	M
2006	84	25/03	L	85	26/03	L	101	10/04	M
2007	14	14/01	E	11	11/01	E	98	8/04	M
2008	20	20/01	E	21	21/01	E	97	6/04	M
2009	63	4/03	L	63	4/03	L	97	6/04	M
2010	66	7/03	L	74	15/03	L	90	31/03	E
Min	12			11			87.0		
Me	41.5			43.0			97.0		
Max	84			85			113.0		
$\bar{x}$	46.2			47.2			98.6		
SD	21.3			21.8			7.2		
V %	46.0			46.2			7.3		
−0.95 % CI	35.6			36.3			89.3		
+0.95 % CI	56.8			58.0			97.7		
**2011**	**42**	**10/02**	**M**	**43**	**11/02**	**M**	**96**	**5/04**	**M**
**2012**	**66**	**8/03**	**L**	**73**	**15/03**	**L**	**94**	**4/04**	**E**

The season starts in 2011 and 2012, as years of model validation are given separately. The *Alnus* and *Corylus* season starts in 1992 and 1996 are excluded from the analysis, because of technical problems

* Cumulative pollen sum (day of the year). Symbol of season type: *E* early; *M* moderate; and *L* late season start. Season starts calculated in the years of model validation (in bold)

In comparison, the *Betula* pollen seasons started on the 99th day of the year (on average, ±7 days), ranging from April 2 to April 15. The seasons defined as moderate prevailed in the studied period. *Betula* pollen seasons are more stable in comparison with *Alnus* and *Corylus* pollen seasons, which is confirmed by the coefficients of variation (7.3, 46 and 46.2 %, respectively).

### Predictive models

The logistic regression model for the start of the *Alnus* pollen season is as follows:

$$P\left( {\text{A}} \right) = \frac{{e^{{ - 3.588 + 0.065 * {\text{Day}} + 0.185 *t5{\text{mean}}}} }}{{1 + e^{{ - 3.588 + 0.065 * {\text{Day}} + 0.185 *t5{\text{mean}}}} }}$$

*P*(A)—probability of *Alnus* pollen season start; Day—day of “local minimum”; *t*_5mean_—5-day running mean of maximum temperature; and *e*—the base of the natural logarithm (about 2.718).

The logistic regression model for the start of the *Corylus* pollen season is as follows:

$$P\left( {\text{C}} \right) = \frac{{e^{{ - 4.155 + 0.074 * {\text{Day}} + 0.215 *t5{\text{mean}}}} }}{{1 + e^{{ - 4.155 + 0.074 * {\text{Day}} + 0.215 *t5{\text{mean}}}} }}$$

*P*(C)–probability of *Corylus* pollen season start; Day–day of “local minimum”; *t*_5mean_—5-day running mean of maximum temperature; and *e*—the base of the natural logarithm (about 2.718).

The logistic regression model for the start of the *Betula* pollen season is as follows:

$$P\left( {\text{B}} \right) = \frac{{e^{{ - 25.413 + 0.273 * {\text{Day}} + 0.352 *t5{\text{mean}}}} }}{{1 + e^{{ - 25.413 + 0.273 * {\text{Day}} + 0.352 *t5{\text{mean}}}} }}$$

*P*(B)—probability of *Betula* pollen season start; Day—day of “local minimum”; *t*_5mean_—5-day running mean of mean temperature; and *e*—the base of the natural logarithm (about 2.718).

The value of *χ* ^2^ statistics (goodness of model fit) (*χ* ^2^ = 18.018 and 22.468, respectively, for *Alnus* and *Corylus; p* < 0.05) indicates that the proposed models contribute something new, because they differ from models including the constant only in significant way (Table 2). The estimation of model parameters for the independent variables and also for intercept indicates their statistically significance (*p* < 0.05) for all studied taxa. In models for *Alnus* and *Corylus*, the odds ratio accepts positive values for all analysed parameters. On the basis of the OR value, it could be calculated that in 10 days from the “local minimum” the chance of probability of the pollen season occurrence increases twice for *Alnus* and 2.1 times for *Corylus*. On the other hand, when the 5-day running mean of maximum temperature increases by 5 °C, the chance of the season start accelerates 2.5 times for *Alnus* and 2.9 times for *Corylus*. The value of *χ* ^2^ statistics obtained for the model constructed for *Betula* pollen season start dates was higher than for *Alnus* and *Corylus* (*χ* ^2^ = 52.821), and it was also statistically significant (*p* < 0.05) (Table 2). On the basis of the OR value, it could be calculated that in 10 days from the “local minimum” the probability of the pollen season occurring increases 15 times, i.e. 7 times more than in the case of *Alnus* and *Corylus*. Moreover, when 5-day running mean temperatures increase by 5 °C, the chance of the season start accelerates 6 times, which was also higher compared to *Alnus* and *Corylus*.

**Table 2:** The estimation of logistic model parameters calculated for the *Alnus*, *Corylus* and *Betula* season onset in Krakow in 1991–2010

Taxon	*Alnus* (*χ* ^2^ = 18.018; *p* = 0.00012)			*Corylus* (*χ* ^2^ = 22.468; *p* = 0.00001)			*Betula* (*χ* ^2^ = 52.821; *p* = 0.0000)		
Statistics	Constant	Day of “local minimum”	5-day running mean of maximum temperature	Constant	Day of “local minimum”	5-day running mean of maximum temperature	Constant	Day of “local minimum”	5-day running mean of mean temperature
Model parameter estimation	−3.588	0.066	0.185	−4.155	0.074	0.215	−25.413	0.274	0.352
Standard error of estimation	0.860	0.02	0.092	0.963	0.021	0.101	6.557	0.074	0.157
*t*(64)	−4.174	3.232	2.005	−4.314	3.553	2.121	−3.876	3.701	2.241
p*	0.00009	0.002	0.049	0.00005	0.0007	0.03	0.0002	0.0004	0.028
−95 % CI	−5.306	0.025	0.0006	−6.077	0.033	0.013	−38.489	0.126	0.039
+95 % CI	−1.871	0.106	0.3692	−2.232	0.116	0.417	−12.336	0.421	0.665
*χ* ^2^ (Wald Statistic)	17.421	10.449	4.019	18.612	12.624	4.498	15.023	13.695	5.024
p**	0.00003	0.001	0.044	0.00002	0.0004	0.03	0.0001	0.0002	0.025
Odds ratio	0.028	1.068	1.203	0.016	1.077	1.239	0.000000000009	1.315	1.422
−95 % CI	0.005	1.025	1.0007	0.002	1.033	1.013	1.92410300000	1.135	1.039
+95 % CI	0.154	1.112	1.447	0.107	1.123	1.517	0.000004389463	1.524	1.945

p*—Calculated for tests of significance; p**—for the Wald statistic

The proposed model for *Alnus* forecasts “lack of the pollen season” in 94 % of cases, and the season start in 43 % of cases, while the model arranged for *Corylus* forecasts “lack of the pollen season” in 93 % of cases and the season start in 50 % of cases (Table 3).

**Table 3:** Classification of correct and incorrect predictions of the *Alnus* and *Corylus* pollen season start in Krakow in 1991–2010

Observed	Predicted—YES (season start)	Predicted—NO (no season start)	% of correct predictions	Predicted—YES (season start)	Predicted—NO (no season start)	% of correct predictions	Predicted—YES (season start)	Predicted—NO (no season start)	% of correct predictions
Taxon	*Alnus*			*Corylus*			*Betula*		
Season start	6	8	42.86	7	7	50.00	51	2	96.23
No season start	3	50	94.34	4	52	92.86	5	15	75.00
Odds ratio: 12.50 %; correct: 83.58 %				Odds ratio: 13.00 %; correct: 84.29 %			Odds ratio: 76.50 %; correct: 90.41 %		

The correctness of prediction indicated that the data used for the model arrangement fitted the models well and stressed the high efficacy of model prediction estimated using the pollen data in 1991–2010. Compared to the models proposed for *Alnus* and *Corylus*, the model for the *Betula* accurately predicted that the season would start in 90.41 % of cases (Table 3).

**Fig. 3:**
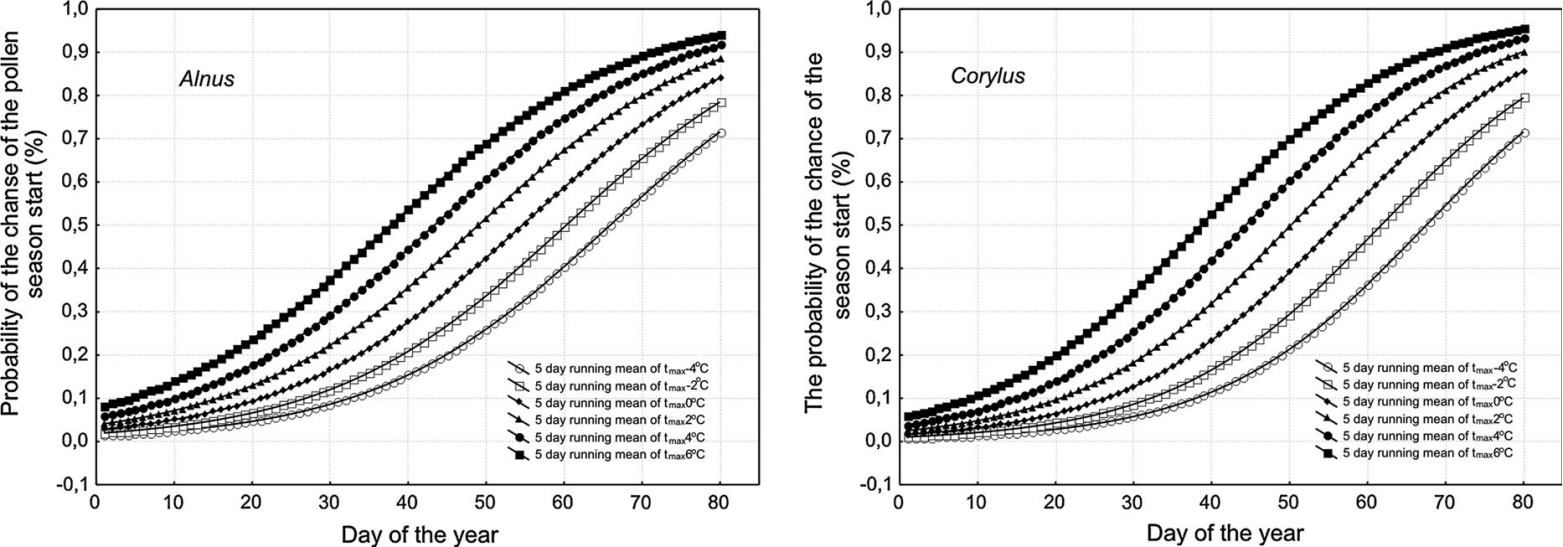
The graphs of logistic regression function presenting the probability of the pollen season occurrence for *Alnus* and *Corylus* at different threshold temperature in Krakow, in 1991–2010. In both cases, 5-day running mean of maximum temperature was calculated (as described in Sect. 2)

Looking at the logistic regression curves of *Alnus* and *Corylus* (Fig. 3), it is clear that in case of both taxa, the curves are highly similar. Introducing the different threshold temperature (range from −4 to 6 °C), the following relation is observed: when the higher 5-day running mean temperature is achieved, the probability of the season start is increased. In addition, a greater chance of earlier season start dates is expected when higher temperatures are achieved.

At the end of January, the chance of the season start at 5-day running mean temperature around −4 °C in comparison with 6 °C decreases below 0.1.

**Fig. 4:**
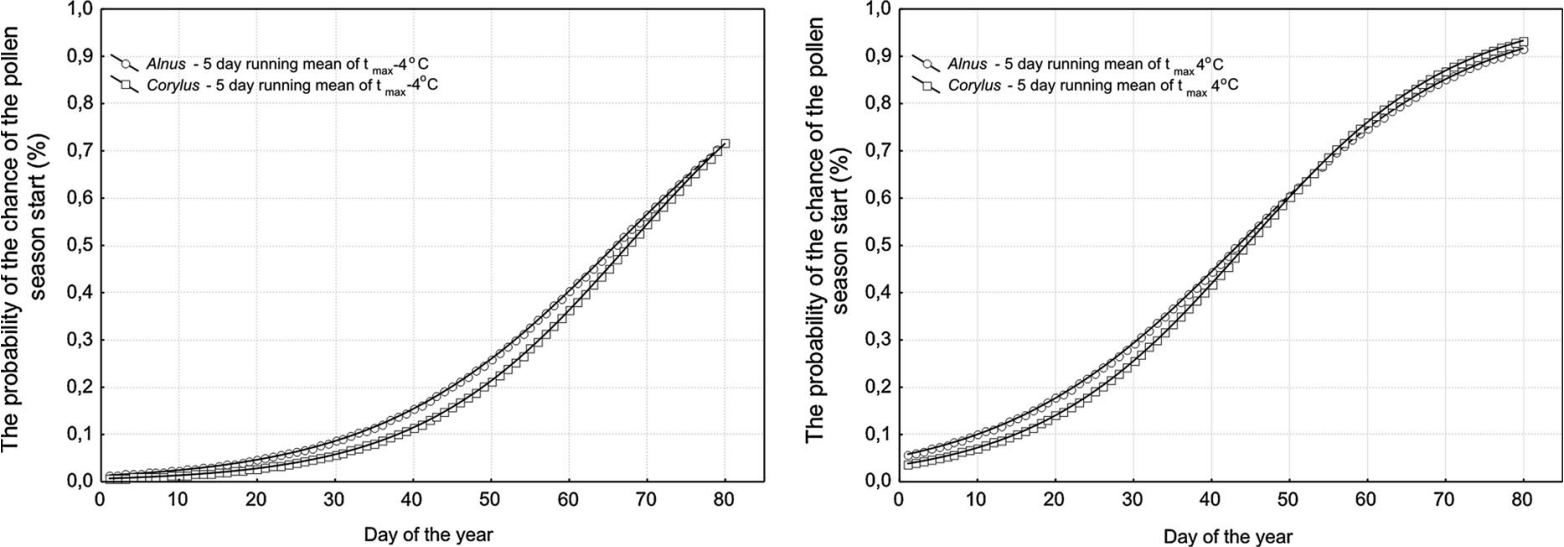
The graphs of logistic regression function presenting the probability of the pollen season occurrence for *Alnus* and *Corylus* at two threshold temperatures (−4 and 4 °C) in Krakow, in 1991–2010. In both cases, the 5-day running mean of maximum temperature was calculated (as described in Sect. 2)

When comparing the reaction of *Alnus* and *Corylus* at two different threshold temperatures (−4 and 4 °C), it was observed that the chance of the *Alnus* pollen season starting is a little higher compared to *Corylus* pollen seasons at lower temperature (−4 °C) (Fig. 4). When the 5-day running mean temperature is positive (4 °C), the function curves cross about 53rd day of the year. The pollen seasons should start on about 66th day of the year in case of *Alnus* and on about 68th day in case of *Corylus*, while at the 5-day running mean temperature = −4 °C, and on about 44th day at temperature 4 °C (for both taxa).

**Fig. 5:**
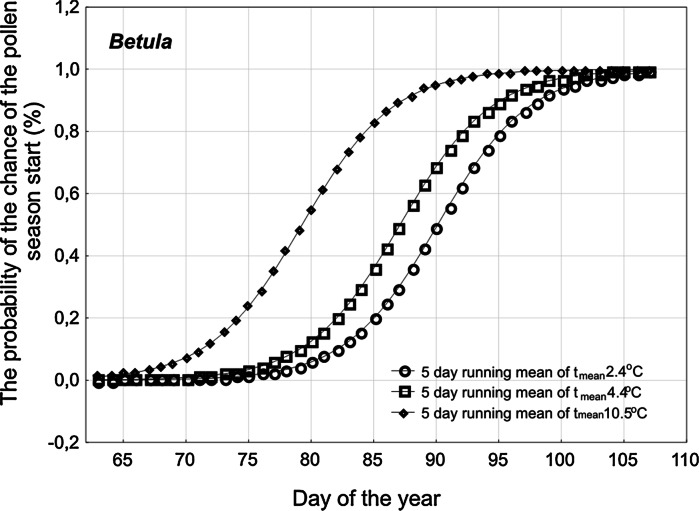
The graph of logistic regression function presenting the probability of the pollen season occurrence for *Betula* at different threshold temperature in Krakow, in 1991–2010. The 5-day mean temperature as 5-day running mean of mean temperature was calculated (as described in Sect. 2)

In the case of *Betula*, the logistic regression curves showed that when higher temperatures are achieved, the probability of the season start increases and a greater chance of an earlier start to the season is expected (Fig. 5). The probability of 0.5 is achieved in the middle of March at a threshold temperature of 10.6 °C (5-day running mean), but at a threshold temperature of 2 °C, this is achieved at the end of March.

### Model verification

The logistic regression models were verified using data from 2011 and 2012 and showed a high efficacy of prediction (Table 4). For *Alnus* and *Corylus*, three “local minima” were considered in 2011, while in 2012, five “local minima” were considered for *Alnus* and six for *Corylus*. In both years, 7 out of 8 cases were predicted correctly, and in the case of *Corylus,* this was 7 out of 9. In 2012, the chance of pollen season start of both taxa was predicted precisely, but in 2011, the season start dates were not predicted by the proposed model. The *Alnus* pollen season started on the 42nd day of the year in 2011. The model failed to predict the season start during 9 days after the “local minimum” that occurred on the 33rd day of the year. A similar situation occurred with the *Corylus* pollen season, which started on the 43rd day of the year in 2011. In 2012, the *Alnus* pollen season started on the 66th day of the year, 6 days after the “local minimum”, the *Corylus* pollen seasons started later, on the 73rd day, as the model predicted. In the case of the *Corylus* pollen season start date, the model correctly predicted the chance of the season occurring after two “local minima” (60 and 68th day of the year), both appeared within 10 days of the observed season start”.

**Table 4:** Regression logistic model verification using data in Krakow, in 2011 and 2012

	2011			2012				
*Alnus*								
Day of “local minimum”	6	25	33	10	18	36	43	60
5-day running mean of maximum temperature on a day of “local minimum”	0.9	−0.1	−0.7	4.3	0.0	−12.4	−8.6	5.6
Probability	0.05	0.12	0.18	0.11	0.08	0.03	0.09	0.80
Forecast—will the season start?	NO	NO	NO	NO	NO	NO	NO	**YES**
Did the season start?	NO	NO	**YES**	NO	NO	NO	NO	**YES**

	2011			2012					
*Corylus*									
Day of “local minimum”	6	25	33	10	18	36	43	60	68
5-day running mean of maximum temperature on a day of “local minimum”	0.9	−0.1	−0.7	4.3	0.0	−12.4	−8.6	5.6	3.7
Probability	0.03	0.09	0.14	0.08	0.06	0.02	0.06	0.82	0.84
Forecast—will the season start?	NO	NO	NO	NO	NO	NO	NO	NO	**YES**
Did the season start?	NO	NO	**YES**	NO	NO	NO	NO	**YES**	**YES**

	2011		2012			
*Betula*						
Day of “local minimum”	80	89	67	82	86	93
5-day running mean of mean temperature on a day of “local minimum”	3.4	5.56	−1.84	8.38	8.68	5.52
Probability	0.09	0.71	0.00	0.50	0.77	0.88
Forecast—will the season start?	NO	**YES**	NO	NO	**YES**	**YES**
Did the season start?	NO	**YES**	NO	NO	**YES**	**YES**

In the case of *Betula* pollen seasons, two “local minima” in 2011 and four “local minima” in 2012 were considered for model verification (Table 4). In all the analysed cases, the model predictions achieved 100 % accuracy. In 2011, the chance of the season start was predicted after the second “local minimum”, and the *Betula* pollen season started 6 days after this. In 2012, an evident decrease in 5-day running mean temperatures occurred on the day immediately before an observed season start. Two “local minima” (86th and 93rd day of the year) were correctly considered as potential days of season start, which was similar to the prediction for *Corylus* pollen season start dates.

## Discussion

In temperate climates, weather conditions occurring at the beginning of the year strongly influence the phenology of trees. These conditions are responsible for plant growth and flowering, but the high variability makes forecasting difficult. For this reason, thermal conditions just before a season should be considered when modelling, because it is almost impossible to make long-range weather predictions for the period January to March, and conditions at this time directly influence the season start.

According to Scamoni (1956), the onset of the *Alnus* pollen season is related to temperatures in the 10 days immediately before the start of a season and pollination is related to warm and dry weather. In the case of *Betula*, the influence of temperatures during this 10-day period before the start of the pollen season was identified by Adams-Groom et al. (2002). This 10-day period is one of the reasons for paying special attention to temperature fluctuations in spring.

In the present study, logistic regression models were built using temperature fluctuations at the beginning of a year. The models included two independent variables: the day of “local minimum” and the value of temperature in the days of “local minimum”. This statistical method was also used by Ribeiro et al. (2007) to define the main pollen season of several taxa. The authors concluded that the fit between the values of the accumulated sum of daily airborne pollen concentrations and the curve calculated using this statistical method was almost perfect.

Heat accumulation after the time of temperature decrease was considered by the author on a “micro-scale” because of the short period between the start of thermal warming in the spring and the onset of pollen seasons. Significant relationships were found between start dates of *Alnus* and *Corylus* pollen seasons and fluctuating temperatures before the season (5-day running mean of maximum temperatures). González-Parrado et al. (2006) calculated heat requirements at *t*_max_ with a threshold temperature of 0 °C. The multiple regression models predicting the *Alnus* pollen season start and including chilling accumulation and heat requirements as independent variables achieved 95 % of accuracy. Piotrowska and Kaszewski (2009) reported that the onset of the *Corylus* pollen season is related to the number of days when *t*_mean_ < 0 °C and cumulated *t*_mean_ in 5-days before a season. Frenguelli et al. (1991) showed that in the Mediterranean area, *Alnus* pollen season start dates depended on *t*_mean_ in the three decades of days before a season. Another study about *Corylus* pollen season start dates in Italy (Frenguelli et al. 1992) reported that thermal conditions in the 10-day period before a season have an impact on the season start provided that *t*_mean_ > 4.5 °C. If mean temperatures do not achieve this threshold value during the period 10 days before the season start, then the season start could be delayed by up to 30–35 days. Similarly, in the present paper, it was documented that at increasing 5-day running mean of maximum temperature, the chance of the season start in case of *Alnus* and *Corylus* increases several times. The response to temperature fluctuations of *Corylus* seems to be a little bit faster.

Start dates of *Betula* pollen seasons in Krakow were found to be more stable than start dates of *Alnus* and *Corylus* pollen seasons in the city. This phenomenon could be explained by the more stable thermal conditions in the months when *Betula* pollen is usually recorded (March, April). As reported by Sarvas (1967), *B. pendula* pollination starts in Poland, Germany, the Czech Republic, Slovakia and southern Finland when 4.1–5.4 % of the mean annual sum of temperature, calculated above the threshold value of 5 °C, is achieved. The author also pointed out that the thermal conditions during the 30-day period before a pollen season determine the beginning and intensity of pollination. The present paper demonstrates that more or less intensive temperature fluctuations occur during the 30 days before a season. The analysis of the plant response in relation to these fluctuations seems to be more objective.

Norris-Hill (1998) presented the highly effective multiple regression models (*R* = 0.996) predicting the *Betula* pollen season onset using *t*_mean_ in February, while Laadi (2001) showed an evident impact of *t*_max_ in a 10-day period in the third decade of February and *t*_min_ at the end of November. In other studies performed in England, Emberlin et al. (1993) found cumulated *t*_mean_ in March as the most important indicator of the *Betula* pollen season start, but the proposed models (multiple regression) achieved only 54.5 % of accuracy. Moreover, if *t*_mean_ in February and March was considered, the models’ efficacy increased up to 61.8 %. The thermal conditions in February were also considered by Bringfelt et al. (1982), both *t*_mean_ and *t*_max_, while Spieksma et al. (1995) indicated that the air temperature during the preceding 4 decades of days before the *Betula* pollen season start is of decisive importance. One of a few examples of dynamic models based on the GDH calculation was presented by Andersen (1991). This model predicted the season start with a 3–5 day precision.

## Conclusions

1. The temperature decrease followed by the temperature increase (named as “local minimum*”*) was found as an indicator of a coming pollen season of *Alnus*, *Corylus* and *Betula*. Basing on this observation, 5-day running means of maximum or mean daily temperature (depending on a studied taxon) were included into the logistic regression models predicting the probability of pollen season start.
2. The estimation of model parameters indicated their statistical significance for all studied taxa; the odds ratio was higher in models for *Betula*, comparing to *Alnus* and *Corylus*.
3. The correctness of prediction indicated that the data used for the model arrangement fitted the models well and stressed the high efficacy of model prediction estimated using the pollen data in 1991–2010. Compared to the models proposed for *Alnus* and *Corylus*, the model for the *Betula* predicted the season start with the highest accuracy.
4. The model verification using data from 2011 to 2012 confirmed a high efficacy of prediction. In case of *Alnus* and *Corylus*, the chance of pollen season start of both taxa was predicted more precisely in 2012, while in case of *Betula,* the model predictions achieved 100 % accuracy in both years.
5. The proposed models seem to be a good tool for aerobiological and medical purposes, expanding the knowledge of the early-pollinating tree pollination and pollen seasons.
